# Cumulative Risk of Type 2 Diabetes in a Working Population: The Japan Epidemiology Collaboration on Occupational Health Study

**DOI:** 10.2188/jea.JE20170093

**Published:** 2018-11-05

**Authors:** Huanhuan Hu, Tohru Nakagawa, Hiroko Okazaki, Chihiro Nishiura, Teppei Imai, Toshiaki Miyamoto, Naoko Sasaki, Makoto Yamamoto, Taizo Murakami, Takeshi Kochi, Masafumi Eguchi, Kentaro Tomita, Satsue Nagahama, Keisuke Kuwahara, Isamu Kabe, Tetsuya Mizoue, Seitaro Dohi

**Affiliations:** 1 Department of Epidemiology and Prevention, National Center for Global Health and Medicine, Tokyo, Japan; 2 Hitachi, Ltd., Ibaraki, Japan; 3 Mitsui Chemicals, Inc., Tokyo, Japan; 4 Tokyo Gas Co., Ltd., Tokyo, Japan; 5 Azbil Corporation, Tokyo, Japan; 6 Nippon Steel & Sumitomo Metal Corporation Kimitsu Works, Chiba, Japan; 7 Mitsubishi Fuso Truck and Bus Corporation, Kanagawa, Japan; 8 YAMAHA CORPORATION, Shizuoka, Japan; 9 Mizue Medical Clinic, Keihin Occupational Health Center, Kanagawa, Japan; 10 Furukawa Electric Co., Ltd., Tokyo, Japan; 11 Mitsubishi Plastics, Inc., Tokyo, Japan; 12 All Japan Labour Welfare Foundation, Tokyo, Japan; 13 Teikyo University Graduate School of Public Health, Tokyo, Japan

**Keywords:** type 2 diabetes, cumulative risk, epidemiology, Japanese

## Abstract

**Background:**

We estimated the cumulative risk of type 2 diabetes from age 30 to 65 years in a large working population in Japan.

**Methods:**

We used data from the Japan Epidemiology Collaboration on Occupational Health Study. Participants (46,065 men and 7,763 women) were aged 30–59 years, free of diabetes at baseline, and followed up for a maximum of 7 years. Incident type 2 diabetes was defined based on fasting and casual glucose, glycated hemoglobin, and current medical treatment for type 2 diabetes. We calculated the sex-specific cumulative risk of type 2 diabetes using the Practical Incidence Estimator macro, which was created to produce several estimates of disease incidence for prospective cohort studies based on a modified Kaplan-Meier method.

**Results:**

During 274,349 person-years of follow-up, 3,587 individuals (3,339 men and 248 women) developed type 2 diabetes. The cumulative risk was 34.7% (95% confidence interval, 33.1–36.3%) for men and 18.6% (95% confidence interval, 15.5–21.7%) for women. In BMI-stratified analysis, obese (BMI ≥30 kg/m^2^) and overweight (BMI 25–29.9 kg/m^2^) men and women had a much higher cumulative risk of type 2 diabetes (obese: 77.3% for men and 64.8% for women; overweight: 49.1% and 35.7%, respectively) than those with BMI <25 kg/m^2^ (26.2% and 13.4% for men and women, respectively).

**Conclusions:**

The present data highlight the public health burden of type 2 diabetes in the working population. There is a need for effective programs for weight management and type 2 diabetes screening, especially for young obese employees, to prevent or delay the development of type 2 diabetes.

## INTRODUCTION

Diabetes, mainly type 2 diabetes, is a major global public health issue, and Asian countries contribute to more than 60% of the world’s diabetic population.^1^^,^^2^ In Japan, there are 10.8 million patients with diabetes.^1^ Lifetime risk of type 2 diabetes has been estimated to determine the risk of developing type 2 diabetes for a general population^3^; however, such data are scarce for the working population. Identifying the cumulative risk of type 2 diabetes during the working lifetime of individuals would provide a better understanding of the development of type 2 diabetes and could urge workers, employers, occupational health professionals, and policy makers to take actions for its prevention.

In Japan, employees are required by law to undergo annual health examinations, which include glycemic measurements. We used this data to estimate the cumulative risk of type 2 diabetes from age 30 to 65 years.

## METHODS

### Study design

The Japan Epidemiology Collaboration on Occupational Health (J-ECOH) Study is an ongoing multicenter health checkup-based cohort study among workers from several companies in Japan.^4^^,^^5^ As of May 2015, 11 participating companies provided health checkup data of their employees, obtained between January 2008 and December 2014 or between April 2008 and March 2015. The study protocol, including the consent procedure, was approved by the Ethics Committee of the National Center for Global Health and Medicine, Japan.

In the present study, the data from the earliest health checkup (mostly carried out in 2008) were regarded as the baseline data; however, if the 2008 dataset contained a large number of missing data, then the data from the 2009 or 2010 (one company each) health checkups were used as the baseline. The outcome was determined using health checkup data from baseline through March 2015.

### Participants

Of 75,857 participants aged 30–59 years who received health checkup during the baseline period, we excluded participants who had a history of diabetes or undiagnosed diabetes at baseline (*n* = 4,924); who had missing information on glucose (*n* = 6,246), glycated hemoglobin (HbA1c; *n* = 1,085), or medical treatment for diabetes (*n* = 407); and who had blood drawn in the non-fasting state (*n* = 7,022). Of the remaining 56,173 participants, we excluded participants who did not attend any subsequent health checkups (*n* = 2,217) and those who attended but did not receive glucose measurements (*n* = 128). A total of 53,828 participants, comprising 46,065 men and 7,763 women, were included for analysis.

### Health checkup

Participants received annual health checkups that included measurements of weight, height, blood pressure, blood glucose, and lipids. The details of health checkups have been described elsewhere.^4^^,^^5^ Body mass index (BMI) was calculated as weight in kilograms divided by height in meters squared. We classified BMI into three groups according to the international overweight and obesity criteria: <25 kg/m^2^, 25 to 29.9 kg/m^2^ (overweight), and ≥30 kg/m^2^ (obesity).^6^

### Outcome

Incident diabetes was identified using data from the health checkups after the baseline examination. Diabetes was defined according to the American Diabetes Association criteria for the diagnosis of diabetes as glycated hemoglobin (HbA1c) ≥6.5%, fasting plasma glucose ≥126 mg/dL, random plasma glucose ≥200 mg/dL, or currently under medical treatment for diabetes.^7^ Individuals without diabetes at baseline who met any of the above conditions in the subsequent checkups were considered to have an incident case of type 2 diabetes.

### Statistical analysis

Characteristics of the study participants were described as means for continuous variables and percentages for categorical variables. Trend association was assessed by assigning ordinal numbers to each BMI group and was tested using a linear regression analysis and the Cochran-Armitage trend test for continuous and categorical variables, respectively.

We calculated sex-specific cumulative risk of type 2 diabetes from age 30 years to age 65 years using the Practical Incidence Estimator macro.^8^ Details of the macro have been extensively described in previous papers.^8^^–^^10^ Briefly, this macro uses age as a time scale of analysis and can produce several estimates of disease incidence (eg, age-specific incidence, cumulative incidence, and reaming lifetime risk) for prospective cohort studies, based on a modified Kaplan-Meier method.^8^ It combines information on participants who entered the cohort study at different ages and takes into account varying duration of follow-up of participants.^8^ All statistical analyses were performed using SAS version 9.4 (SAS Institute, Cary, NC, USA).

## RESULTS

The characteristics of participants by sex and BMI groups are shown in Table 1. Among cohort participants, 24.7% of men and 11.5% of women had a BMI of 25 to 29.9 kg/m^2^, and 3.2% of men and 2.7% of women had a BMI ≥30 kg/m^2^. For both sexes, the waist circumference, systolic and diastolic blood pressure, fasting plasma glucose, HbA1c, and triglyceride and low-density lipoprotein-cholesterol levels increased with BMI (*P* for trend <0.001). The prevalence rates of hypertension and dyslipidemia were higher in people with higher levels of BMI (*P* for trend <0.001). During 274,349 person-years of follow-up, 3,587 individuals (3,339 men and 248 women) developed type 2 diabetes. The crude incidence rate of type 2 diabetes (per 1,000 person-years) was 14.2 for men and 6.4 for women. Sex- and age-specific incidence rates of type 2 diabetes are provided in eTable 1.

**Table 1.** Baseline characteristics by sex and baseline BMI

	Men			*P* for trend	Women			*P* for trend
	
	BMI <25 kg/m^2^	BMI ≥25 to <30 kg/m^2^	BMI ≥30 kg/m^2^		BMI <25 kg/m^2^	BMI ≥25 to <30 kg/m^2^	BMI ≥30 kg/m^2^	
*N*	33,212	11,387	1,466		6,662	891	210	
BMI, kg/m^2 a^	22.1 (1.9)	26.8 (1.3)	32.4 (2.6)		20.6 (2.1)	26.9 (1.4)	32.7 (2.7)	
Age, years^a^	45.4 (7.9)	45.6 (7.6)	42.9 (7.0)	<0.001	43.9 (7.5)	45.9 (7.6)	44.7 (7.2)	<0.001
WC, cm^a^	80.0 (6.0)	91.0 (5.0)	103.5 (7.0)	<0.001	73.5 (7.0)	88.0 (5.8)	98.8 (7.1)	<0.001
SBP, mm Hg^a^	119.6 (14.5)	126.6 (14.2)	132.8 (14.8)	<0.001	113.7 (15.5)	124.6 (14.5)	132.2 (15.2)	<0.001
DBP, mm Hg^a^	75.8 (10.2)	80.8 (10.0)	84.6 (10.2)	<0.001	70.7 (10.3)	77.7 (9.8)	82.0 (10.6)	<0.001
Hypertension, %^b^	15.7	29.5	46.9	<0.001	8.2	20.8	39.1	<0.001
Anti-hypertension treatment, %^c^	43.4	48.6	49.8	<0.001	39.3	48.1	34.2	<0.001
FPG, mg/dL^a^	96.2 (8.7)	99.1 (9.0)	100.0 (9.7)	<0.001	90.5 (7.8)	94.5 (8.6)	97.6 (10.1)	<0.001
HbA1c, %^a^	5.4 (0.4)	5.6 (0.4)	5.7 (0.4)	<0.001	5.4 (0.3)	5.6 (0.4)	5.7 (0.4)	<0.001
TG, mg/dL^a^	116.8 (84.0)	160.2 (109.2)	174.8 (125.4)	<0.001	71.8 (40.9)	103.6 (58.6)	117.0 (67.5)	<0.001
LDL-C, mg/dL^a^	118.2 (29.0)	129.1 (29.4)	133.7 (31.5)	<0.001	111.7 (28.8)	130.7 (30.6)	131.9 (30.3)	<0.001
HDL-C, mg/dL^a^	59.0 (14.7)	51.1 (11.6)	47.7 (9.8)	<0.001	71.3 (15.4)	59.9 (12.9)	56.3 (13.7)	<0.001
Dyslipidemia, %^d^	40.0	65.1	75.4	<0.001	20.2	48.2	53.8	<0.001
Lipid-lowering treatment, %^e^	8.5	11.1	15.8	<0.001	12.8	13.8	12.4	<0.001
Smoking, %	41.5	41.8	46.1	0.006	11.1	10.9	13.9	0.437

BMI, body mass index; DBP, diastolic blood pressure; FPG, fasting plasma glucose; HbA1c, glycated hemoglobin; HDL-C, high-density lipoprotein cholesterol; LDL-C, low-density lipoprotein cholesterol; SBP, systolic blood pressure; TG, triglyceride; WC, waist circumference.

^a^Mean (standard deviation).

^b^Hypertension was defined as SBP ≥140 mm Hg, DBP ≥90 mm Hg, or as receiving medical treatment for hypertension, based on the criteria of The Japanese Society of Hypertension Guidelines for the Management of Hypertension.

^c^The denominator is the total number of people with hypertension.

^d^Dyslipidemia was defined as TG ≥150 mg/dL, LDL-C ≥140 mg/dL, HDL-C <40 mg/dL, or as receiving medical treatment for dyslipidemia, based on the criteria of the Japan Atherosclerosis Society.

^e^The denominator is the total number of people with dyslipidemia.

**Figure 1.**
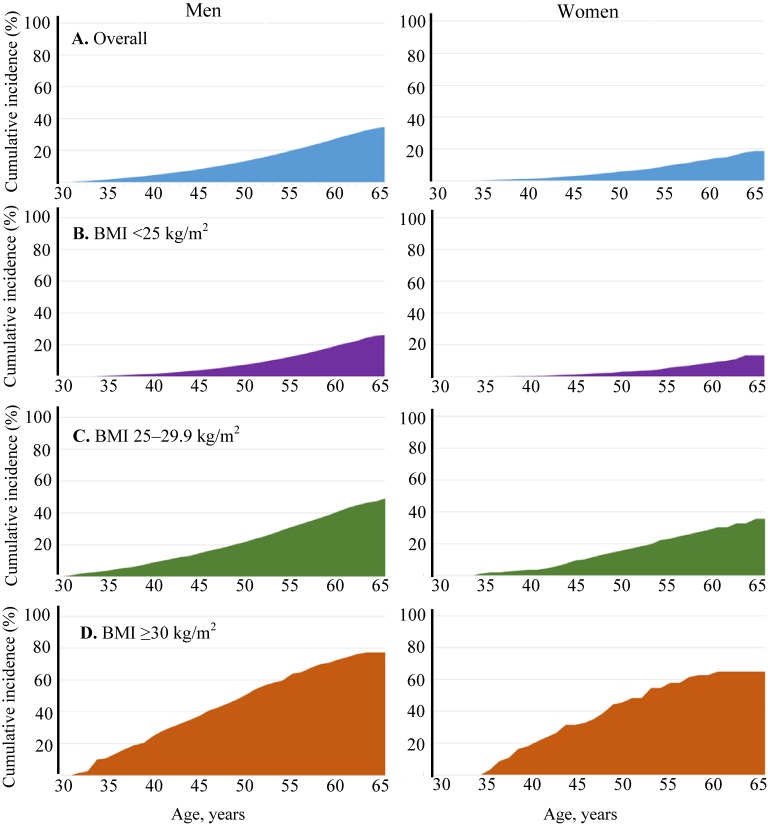
Cumulative risk of diabetes in individuals aged 30 years. A. Overall; B. BMI <25 kg/m^2^; C. BMI 25–29.9 kg/m^2^; D. BMI ≥30 kg/m^2^.

Figure 1 shows the cumulative incidence functions in individuals aged 30 years. The cumulative risk of developing type 2 diabetes from age 30 years to age 65 years was 34.7% (95% confidence interval, 33.1–36.3%) for men and 18.6% (95% confidence interval, 15.5–21.7%) for women. Stratification by BMI showed that people with a BMI ≥30 kg/m^2^ had much higher cumulative risk of type 2 diabetes (77.3% for men and 64.8% for women) than people with a BMI of 25 to 29.9 kg/m^2^ (49.1% for men and 35.7% for women) or people with a BMI <25 kg/m^2^ (26.2% for men and 13.4% for women). We estimated that more than 1 in 3 obese men aged 30 years would develop type 2 diabetes by age 45 years.

## DISCUSSION

Based on a large-scale cohort study among Japanese employees, we estimated that one-third of men and one-fifth of women at age 30 years will develop type 2 diabetes by age 65 years. Furthermore, the cumulative risk of type 2 diabetes substantially increased among people with a BMI ≥25 kg/m^2^. To our knowledge, this is the first study to estimate the cumulative risk of type 2 diabetes from age 30 years to age 65 years in the working population.

Similar to the present study, high lifetime risk of diabetes has been reported in the USA (40% at age 20 years),^11^ Australia (31% at age 45 years),^12^ and the Netherlands (31% at age 45 years).^3^ However, our study differed from previous studies in design, target population, and target period (cumulative risk from age 30 to 65 years in the present study compared to overall lifetime risk in previous studies). Furthermore, we defined incident type 2 diabetes based on fasting and casual glucose, HbA1c, and current medical treatment for diabetes, while researchers of previous studies did not use HbA1c^3^^,^^12^ or only used self-reported diabetes.^11^ Therefore, caution should be exercised when comparing the current estimate with those reported previously.

Previous studies have reported sex differences in type 2 diabetes prevalence in the Japanese population.^13^^,^^14^ One meta-regression analysis of 160,000 Japanese adults showed that the age-standardized diabetes prevalence was 9.3% and 6.6% among men and women, respectively, in 2010.^13^ We also found that the cumulative risk of type 2 diabetes among women (18.6%) was lower compared with men (34.7%). This may be partly ascribed to the lower prevalence rates of risk factors for type 2 diabetes (eg, obesity, smoking, hypertension, and dyslipidemia) in women than in men. Further efforts focusing on these risk factors may not only reduce diabetes risk in the working population but also narrow the gender gap in diabetes risk.

Obese workers (BMI ≥30 kg/m^2^) were at markedly increased risk of developing type 2 diabetes; more than 1 in 3 obese men at age 30 years were predicted to develop type 2 diabetes by age 45 years (Figure 1). Lifetime risk of type 2 diabetes increases with increasing BMI.^3^^,^^15^ Obesity reduces the time lived with normal glucose metabolism.^3^ These findings underscore the importance of weight management in the prevention of type 2 diabetes.

The strengths of our study include its large sample size and sufficient number of type 2 diabetes events. In addition, we used a comprehensive assessment of multiple measures for the diagnosis of incident type 2 diabetes. Several limitations also warrant attention. First, the present findings from a working population may not be generalizable to a wider population. Given the lower sex- and age-specific prevalence of overweight/obesity (BMI ≥25 kg/m^2^) in the present study than that in the National Health and Nutrition Survey,^16^ higher cumulative incidence would be expected in the general population. Second, the cumulative risk of developing type 2 diabetes would be somewhat overestimated due to the lack of adjustment for competing risks, such as death. Third, the estimates of cumulative risk are subject to birth-cohort effects; thus, caution should be exercised when age-specific incidence rates are rapidly changing over time. Finally, we could not discriminate between type 1 and type 2 diabetes. However, since type 1 diabetes is rare in people aged 30 years and older, we expect that virtually all incident cases in this cohort correlate with type 2 diabetes.

The present data highlight the public health burden of type 2 diabetes among the working population. There is a need for effective weight management and type 2 diabetes screening programs, especially for young obese employees, to prevent or delay the development of type 2 diabetes.

## APPENDIX A. SUPPLEMENTARY DATA

eTable 1. Incidence rates of type 2 diabetes by sex and age groups
