# Supplementary material for: Cumulative Risk of Type 2 Diabetes in a Working Population: The Japan Epidemiology Collaboration on Occupational Health Study
Source: J Epidemiol. 2018 Nov 5;28(11):465–9. doi: 10.2188/jea.JE20170093 (PMC6192974; doi:10.2188/jea.JE20170093)
Supplement: Supplementary file 1 [file je-28-465-s001.pdf]

eTable 1. Incidence rates of type 2 diabetes by sex and age groups

Age group	Cases	Total people	Person-years	Incidence (/1,000 person-years)
Men				
30–34 years	108	3,335	18,618	5.8
35–39 years	406	9,063	50,670	8.0
40–44 years	581	9,998	53,876	10.4
45–49 years	633	8,073	42,636	14.8
50–54 years	846	7,927	39,831	21.2
55–59 years	765	7,669	29,751	25.7
Women				
30–34 years	10	619	3,228	3.1
35–39 years	23	1,884	10,262	2.2
40–44 years	55	1,771	9,191	6.0
45–49 years	50	1,389	7,175	7.0
50–54 years	62	1,111	8,445	7.3
55–59 years	48	989	3,660	13.1
